# Purifying synthetic high-strength wastewater by microalgae *chlorella vulgaris* under various light emitting diode wavelengths and intensities

**DOI:** 10.1186/2052-336X-11-8

**Published:** 2013-06-13

**Authors:** Zhigang Ge, Hui Zhang, Yuejin Zhang, Cheng Yan, Yongjun Zhao

**Affiliations:** 1 College of Biological Chemical Science and Engineering, Jiaxing University, Jiaxing, P.R. China; 2 Department of Environmental Science and Engineering, Fudan University, Shanghai, P.R. China

**Keywords:** *Chlorella vulgaris*, Economic efficiency, High carbon loading, High nitrogen loading, Light intensity, Light wavelength

## Abstract

The high-strength wastewater is now well known as a threat to the natural water since it is highly possible to arouse water eutrophication or algal blooms. The effects of various light emitting diode wavelengths and intensities on the microalgae biological wastewater treatment system was studied in this research. The various nutrient removals and economic efficiencies represented similar variation trends, and these variations under both high C and N loading treatments were similar too. The order for microalgae *C*. *vulgaris* reproduction in terms of dry weight and nutrient removal efficiency both were red > white > yellow > blue, under high carbon and nitrogen loading treatments, indicating that the red light was the optimum light wavelength. Furthermore, considering the optimal light intensity in terms of nutrient removal efficiency was 2500 and 2000 μmol/m^2^•s, while in terms of economic efficiency was 1000, 1500 and 2000 μmol/m^2^•s. Therefore, the optimum light intensity was found to be 2000 μmol/m^2^•s. In addition, the optimal experimental illumination time was determined as 120 h. The *Chlorella vulgaris* microalgae biological wastewater treatment system utilized in this research was able to purify the high-strength carbon and nitrogen wastewater effectively under optimum light wavelength and intensity.

## Background

The high-strength wastewater has attracted increasing interests over the past decades owing to its significant effects on water bodies [1]. For instance, the fermentation slurry is rich in carbon (C), nitrogen (N), phosphorus, and other nutrients and highly possible to cause water eutrophication and algal blooms [2,3]. Another kind of typical high-strength wastewater is chemical fertilizer agricultural wastewater which has been reported as one of the principal sources of non-point source pollution [4,5]. It is able to greatly cause surface-water eutrophication and groundwater nitrate enrichment [4]. Therefore, there is an urgent need to develop a bio-system to effectively treat high-strength wastewater. The existing centralized wastewater treatment systems based on the packed-bed biofilm reactors or up-flow anaerobic sludge blanket reactors are not practicable in rural areas of China since their high construction costs and land requirements [6,7]. Nonetheless, the stabilization ponds based on microalgae biological wastewater treatment system has attracted increasing interest due to its high nutrient removal efficiency, low construction costs, and freedom from spatial restrictions [8].

A lot of literatures suggested that the microalgae can efficiently absorb nutrient elements from high-strength wastewater by virtue of their extremely high photosynthetic efficiency [9]. Kumar *et al*. [10] reported that treating digested piggery effluent by *Chlorella vulgaris* could achieved 100% total phosphorus (TP) and 78% NH_4_^+^-N removal efficiency. Lim *et al*. [11] reported that *C*. *vulgaris* has great potential for bioremediation of agro-industrial high-strength wastewater such as rubber effluent and palm oil mill effluent. Phang and Chu [12] reported that *C*. *vulgaris* was shown to be a versatile microalga that is able to grow under various harsh conditions (e.g. high NaNO_3_ or NH_4_Cl levels) and tolerant to high levels of heavy metals, especially Mn, Cr, Zn and Cd. Furthermore, in the process of purifying high-strength wastewater, the wastewater itself also has the potential to be an economic culture medium for *C*. *vulgaris*, which can produce some types of value-added products, since it can be freely obtained and possesses the nutrients required for microalgae growth [13,14]. Arroyo *et al*. [15] indicated that *C*. *vulgaris* showed great potentials as future industrial bioenergy producers due to its robustness, high growth rate, and high oil content, and mixotrophic culturing condition. Ryckebosch *et al*. [8] suggested that high-strength wastewater were able to be utilized as an inexpensive nutrient medium for culturing certain species of microalgae for harvesting as potentially valuable microalgae biomass or metabolic product.

However, the microalgae biological wastewater treatment system always suffers from bad light intensities and wavelengths [16]. In the open air, varying illumination intensities are likely to inhibit microalgae growth because of a shortage in light energy, e.g., very low light intensities during rainy days, or the photoinhibition caused by excessive irradiance, e.g., very high light intensities at noontimes during summer. More importantly, lighting utilization efficiency significantly affects the overall microalgae reproduction process. Indeed, the microalgae require optimal illumination to achieve the maximum photosynthetic rate and nutrient removal efficiency economically [17]. Consequently, using artificial light sources to culture microalgae indoor is an alternative solution. The light wavelength and intensity of artificial light sources are important factors for microalgae growth in biological wastewater treatment systems [17]. The ordinary light sources, such as filament or fluorescent lamps, are less economical and efficient for microalgae metabolism than the light sources with specific narrow bands [18,19]. This is because the ordinary light sources are only a combination of efficient and inefficient light spectra for microalgae growth or even emit spectra outside of the absorption band of microalgae chlorophyll pigments [20]. Therefore, the light emitting diode (LED) is considered to be the optimal light source for microalgae biological wastewater treatment systems because it had the characteristics of narrow-band wavelength and cost-effective power consumption [17]. Consequently, the light wavelength and intensity are both essential factors to microalgae biological wastewater treatment system. For instance, Matthijs *et al*. [21] reported that the monochromatic red LED light was able to support the growth of microalgae *Chlorella spp*., while the partial exposure to blue LED light did not maintain the microalgae reproduction. However, the effects of various LED light wavelengths and light intensities on the treatment of high-strength wastewater by microalgae biological wastewater treatment system remain largely unknown.

This research focused on the responses of the microalgae biological wastewater treatment system to various LED light wavelengths and intensities in terms of the produced dry weight (DW) of *C*. *vulgaris*, the nutrient removal efficiencies of microalgae, and the economic efficiencies for synthetic wastewater purification, under synthetic high C and N loading wastewater. The optimal light wavelength was determined and analyzed to predict and explain the performance of the microalgae biological wastewater treatment system under high-strength wastewater loading. The optimum light intensity was confirmed by analyzing the nutrient removal economic efficiencies under various light intensities.

## Materials and methods

The *C*. *vulgaris* (FACHB-31) microalgae strain was purchased from FACHB-Collection, Institute of Hydrobiology, Chinese Academy of Sciences. The LEDs used in this study had widths of 26 mm and lengths of 600 mm; purchased from Canal Optoelectronic Technology Co., Ltd, P.R. China (Table 1). The LEDs with various light intensities were customized. For health and safety reasons, as well as for comparison of the parallel experiments, the synthetic high-strength wastewater was utilized in this research. It was a modification of Organisation of Economic Co-operation and Development (OECD) standard wastewater [22]. There were two experimental categories: 600 g/m^3^ glucose, 100 g/m^3^ carbamide, 15 g/m^3^ NaH_2_PO_4_, 1.5 g/m^3^ KH_2_PO_4_, 4 g/m^3^ CaCl_2_, and 2 g/m^3^ MgSO_4_ for high C loading treatment; 200 g/m^3^ glucose, 300 g/m^3^ carbamide, 15 g/m^3^ NaH_2_PO_4_, 1.5 g/m^3^ KH_2_PO_4_, 4 g/m^3^ CaCl_2_, and 2 g/m^3^ MgSO_4_ for high N loading treatment. Therefore, the influent wastewater concentrations were: chemical oxygen demand (COD) 603.27±11.38 mg/L, total nitrogen (TN) 53.82±7.21 mg/L, and TP 5.08±0.69 mg/L for high C loading treatment; COD 208.16±12.05 mg/L, TN 154.82±8.75 mg/L, and TP 5.13±0.64 mg/L for high N loading treatment. The pH level was adjusted to 6.50±0.15 using H_2_SO_4_. The mean value of the oxidation reduction potential (ORP) of wastewater was 60.25±8.40 mV. All treatments were performed in quadruplicate.

**Table 1:** The characteristics of LEDs

**Light source**	**Light wavelengths (nm)**	FWHM*	**Energy consumption (W) in various light intensities (μmol/m**^**2**^**•s)**					
			**500**	**1000**	**1500**	**2000**	**2500**	**3000**
Red	640–680	660	0.95	2.07	3.91	4.96	8.74	10.49
White	380–670	-	0.68	2.01	4.42	6.42	8.42	10.41
Yellow	590–600	595	4.35	6.93	10.41	11.91	14.41	16.92
Blue	460–470	465	0.96	3.90	8.52	12.52	16.52	20.51

*FWHM: full width at half maximum.

### Experimental procedure

The experiments were conducted by 1000 mL Erlenmeyer flask containing 400 mL synthetic wastewater and 200 mL *C*. *vulgaris* suspension which initial DW was 90.55±8.75 mg/L. All treatments were maintained in an illuminating incubator (SPX-400I-G, Boxun Industry & Commerce Co., Ltd, P.R. China) at a temperature of 25.0±0.5°C and a 12-hour light–dark cycle (light period was between 8:00 AM and 8:00 PM). The LEDs were installed as light sources in the illuminating incubator. All treatments were sampled and analyzed daily at 2:00 PM during the 8 d/192 h experimental period. In addition, the artificial shaking was performed to the flasks daily prior to sampling.

The optimal light wavelengths for microalgae DW and nutrient removal efficiency were determined by exposing the treatments to red, white, yellow and blue light at the constant light intensity of 2500 μmol/m^2^•s. Then, the treatments were illuminated by the optimal light wavelengths under light intensities 500, 1000, 1500, 2000, 2500 and 3000 μmol/m^2^•s to determine the optimum light intensity range.

#### Sampling and analyses

The DW of *C*. *vulgaris* was measured by the following procedures: first, 15 mL culture suspensions were filtered using glass microfiber filter (GF/C, Whatman, USA); second, the filter with attached *C*. *vulgaris* cell was dried at 100°C for 24 h and then cooled to room temperature in a desiccator; finally, DW was determined from the difference between the filter weights before and after filtration. The filtrates of the cultures were analyzed for COD, TN and TP using closed reflux titrimetric method, persulfate method and ascorbic acid method, respectively [23]. The pH and ORP were measured by a pH meter (Orion 250 Aplus ORP Field Kit, USA). The light intensity was measured by a waterproof light meter inside the medium (CEM, DT-1308, Shenzhen Everbest Machinery Industry Co., Ltd, P.R. China).

The nutrient removal efficiency was calculated as follows:

(1) $$R=\left(1-\frac{C_{i}}{C_{0}}\right)\times 100$$

where *R* is the nutrient removal efficiency (%), *C*_*0*_ and *C*_*i*_ are the nutrient concentrations in the initial wastewater and the culture filtrates (mg/L), respectively.

The economic efficiency for nutrient removal efficiency of synthetic wastewater was calculated as follows:

(2) $$E_{\mathrm{nutrient}}=\frac{R}{\mathrm{kTP}}$$

Where E_nutrient_ is the economic efficiency for nutrient removal efficiency (USD), *R* is the nutrient removal efficiency (%) in Eq. 1, *k* is the cost per unit of energy consumption (USD/kW•h), *T* is the actual illumination time (h) and *P* is the LED energy consumption during the actual illumination time (W). The relationships between energy consumption and light intensity of various LED light wavelengths were shown in Table 1.

### Statistical analyses

All statistical analyses were performed by SPSS software [24]. The different effects of variation in light wavelengths and intensities on microalgae reproduction and nutrient removal efficiency were tested by ANOVA. Duncan’s multiple range tests was used to further assess differences among light wavelengths that were significant in ANOVA. A probability level of P= 0.05 was used as the threshold for significance.

## Results

### Variation of physicochemical properties

The variation trends of pH and ORP values of *C*. *vulgaris* suspension under all light wavelengths treatments for both high C and N loading treatment were similar. The pH values of *C*. *vulgaris* suspension culture increased smoothly through the experimental period from an initial value of 6.50±0.15 to 8.32±0.57, 8.25±0.81, 8.49±0.51 and 8.47±0.64 under the treatment of red, white, yellow and blue light wavelength, respectively. Su *et al*. [25] reported a similar result, who observed that the pH values increased from 6.5 to 8.5 via purifying soybean processing wastewater by *Chlorella pyrenoidosa*. This increase was attributed to the decreased dissolved carbon dioxide concentration and the removal of organic nutrients during the process of the microalgae photosynthesis [26]. The ORP values smoothly decreased from 60.25±8.40 to 50.84±7.21, 53.26±6.95, 51.56±6.34 and 51.36±6.72 mV under the treatment of red, white, yellow and blue light wavelength, respectively, during the experimental period. This was because the accumulation of metabolic products and the consumption of nutrients in the synthetic wastewater.

#### Optimal light wavelengths

Table 2 demonstrated the microalgae DW with various light wavelengths under the constant light intensity of 2500 μmol/m^2^•s. The results for high C and N loading treatments were similar. The red light wavelength achieved significantly higher (*P*<*0*.*05*) microalgae DW than the rest of the light wavelengths, whereas the blue light wavelength achieved significantly lower (*P*<*0*.*05*) microalgae DW than the rest of the light wavelengths. Therefore, the order for microalgae *C*. *vulgaris* reproduction in terms of DW was red > white > yellow > blue under both high C and N loading treatments, indicating that the red light was the optimum light wavelength for microalgae *C*. *vulgaris* reproduction [27,28]. These results were similar to the literatures. Matthijs *et al*. [21] also reported that the red light was optimal light wavelength for *Chlorella pyrenoidosa* culture.

**Table 2:** **Mean values** ± **SD of the microalgae DW and the nutrient removal efficiencies under various light wavelengths at a constant light intensity of 2500** μ**mol**/**m**^**2**^•**s**

**Items**	**Light wavelengths**	**Dry weight (mg**/**L)**	**Nutrient removal efficiency (%)**		
			**COD**	**TN**	**TP**
High C loading	Red	231.74^a^±13.62	82.12^a^±11.28	76.04^a^±8.39	57.88^a^±7.43
	White	210.53^b^±14.85	70.31^b^±5.29	61.31^b^±5.79	29.25^b^±5.32
	Yellow	174.92^c^±17.53	42.17^c^±3.62	35.72^c^±4.06	20.51^c^±3.84
	Blue	96.67^d^±13.81	11.55^d^±3.74	17.35^d^±3.92	11.93^d^±2.41
High N loading	Red	248.69^a^±18.74	81.90^a^±8.26	81.16^a^±7.04	52.72^a^±7.79
	White	207.46^b^±16.38	67.49^b^±4.68	60.27^b^±5.38	28.91^b^±3.57
	Yellow	160.75^c^±15.87	39.72^c^±5.26	38.48^c^±5.46	21.14^c^±4.01
	Blue	94.84^d^±14.79	13.04^d^±3.62	14.32^d^±2.39	13.89^d^±3.51

a, b, c, d: Values with different superscript letters in the same column indicate significant differences at P = 0.05, according to Duncan’s multiple range tests.

Table 2 shows the nutrient removal efficiency of microalgae with various light wavelengths under the constant light intensity of 2500 μmol/m^2^•s. The results for high C and N loading treatments were similar. The removal efficiency of red light wavelength was significantly higher (P<0.05) than the rest of the light wavelengths, whereas that of blue light wavelength was significantly lower (P<0.05) than the rest of the light wavelengths. So, the order for microalgae *C*. *vulgaris* nutrient removal efficiency was red > white > yellow > blue under both high C and N loading treatments. Therefore, the red light was the optimum light wavelength for the nutrient removal efficiency. As show in Table 2, COD, TN, and TP removal efficiency achieved 82.12±11.28%, 76.04±8.39%, and 57.88±7.43%, respectively, for high C loading and 81.90±8.26%, 81.16±7.04% and 52.72±7.79%, respectively, for high N loading. The effects of microalgae biological wastewater treatment system in this research were much better than that in the literatures. Yang *et al*. [29] found that the maximal COD removal efficiency was only 71.2% when cultivating *C*. *pyrenoidosa* with cassava ethanol fermentation under continuous polychromatic wavelengths. Bhatnagar *et al*. [30] also reported that only 30% TP removal efficiency was achieved when treating municipal wastewaters by *Chlorella minutissima* in an oxidation pond. These phenomena indicated that the optimal light wavelength, rather than ordinary light, was able to achieve higher nutrient removal efficiency [31].

### Optimal light intensity

#### Time course of microalgae growth and nutrient removal efficiency under various light intensities

**Figure 1:**
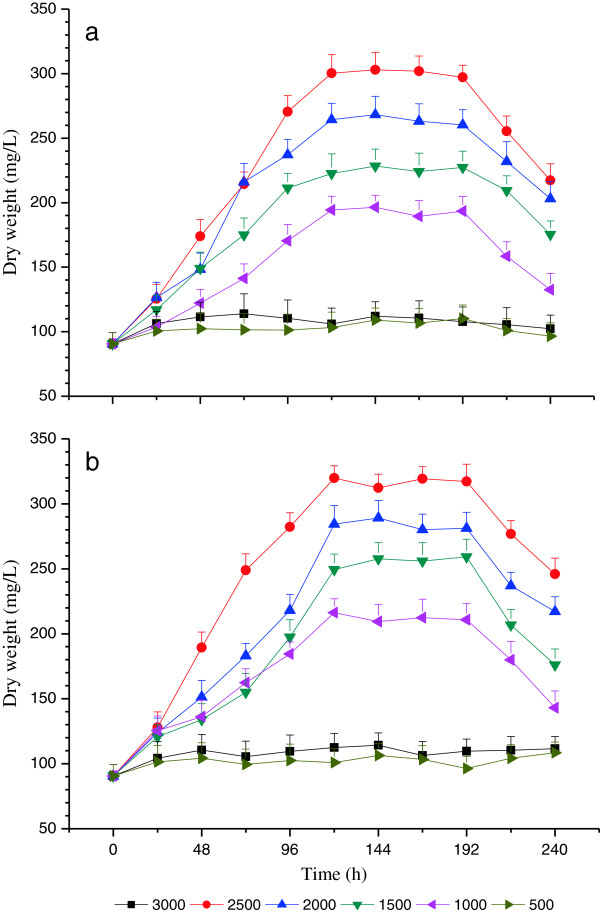
**Time course of microalgae growth with red light wavelength under various light intensities: (a) High C loading and (b) High N loading.**

Time course of microalgae growth with red light wavelength under various light intensities (i.e., 500, 1000, 1500, 2000, 2500 and 3000 μmol/m^2^•s) at high C and N loading treatment are shown in Figure 1. The variation trends of microalgae growth were similar under high C and N loading treatment. But the DW values under 500 and 3000 μmol/m^2^•s were much lower than others during the experimental period. The 500 μmol/m^2^•s was too low to maintain the growth of microalgae. The insufficient light intensity resulted in biomass loss and slower growth rates, as microalgae consumed carbohydrates and oxygen during photorespiration, but were unlikely to cause fatal damage. While, the light intensity of 3000 μmol/m^2^•s was too high to avoid photoinhibition. The excessive light intensity was able to damage or kill microalgae, because it overloaded their photosystems and could even bleach out the pigments [32,33].

**Figure 2:**
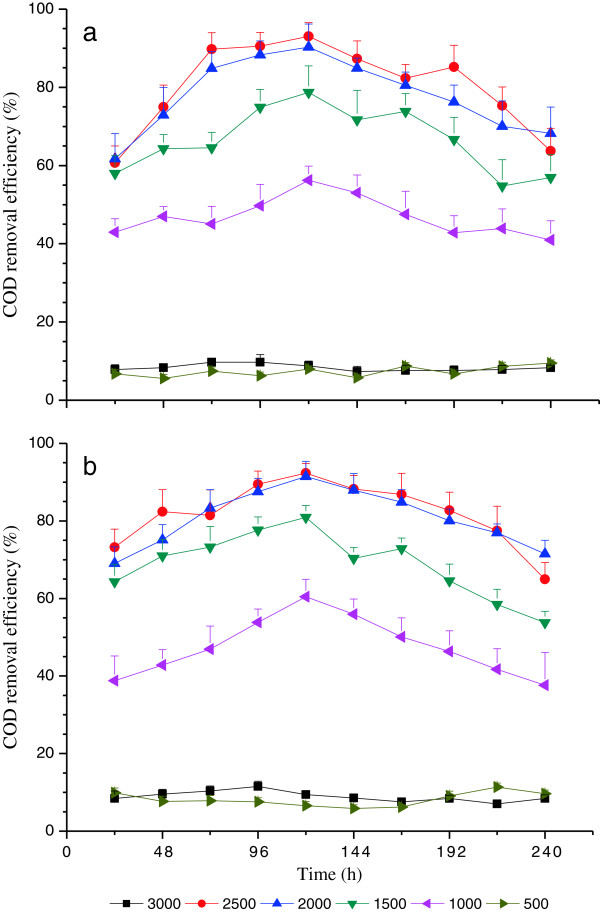
**Time course of COD removal efficiency with red light wavelength under various light intensities: (a) High C loading and (b) High N loading.**

**Figure 3:**
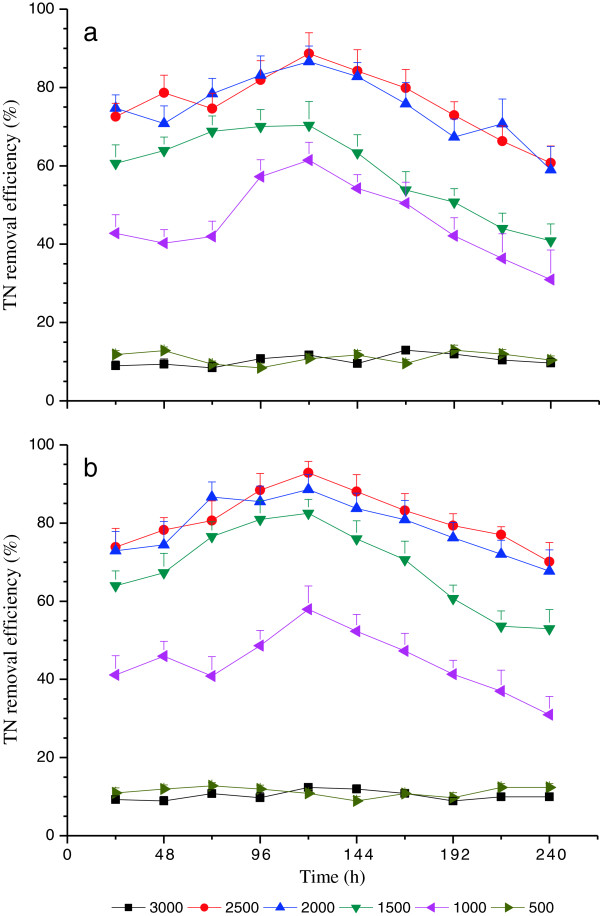
**Time course of TN removal efficiency with red light wavelength under various light intensities: (a) High C loading and (b) High N loading.**

**Figure 4:**
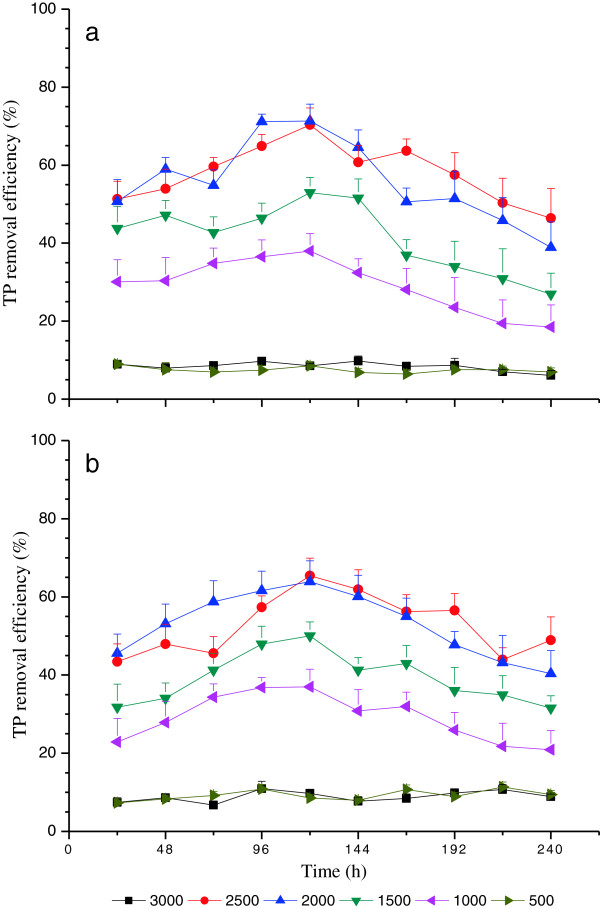
**Time course of TP removal efficiency with red light wavelength under various light intensities: (a) High C loading and (b) High N loading.**

Time course of nutrient removal efficiency with red light wavelength under various light intensities (i.e., 500, 1000, 1500, 2000, 2500 and 3000 μmol/m^2^•s) at high C and N loading treatment are shown in Figures 2, 3 and 4. The removal efficiencies of COD, TN and TP demonstrated similar variation trends, and the variation trends of nutrient removal were also similar under high C and N loading treatments. This is in agreement with the variation of DW curves in terms of time course (Figure 1). The nutrient removal efficiencies under 500 and 3000 μmol/m^2^•s were much lower compared with others sine the 500 μmol/m^2^•s was insufficiently low to maintain the metabolic process of microalgae, whereas 3000 μmol/m^2^•s was too high to avoid photoinhibition [34].

The mean values of removal efficiencies of COD, TN and TP demonstrated similar variation trends and the variation trends of nutrient removal were also similar under high C and N loading treatments (Table 3). There were no significant differences (P>0.05) between the nutrient removal efficiency of 500 and 3000 μmol/m^2^•s light intensity treatments, while they were both significantly lower (P<0.05) than the other light intensity treatments (Table 3). Furthermore, there were no significant differences (P>0.05) between the light intensity treatment of 2500 and 2000 μmol/m^2^•s, but they were both significantly higher (P<0.05) than the light intensity 1500 μmol/m^2^•s (Table 3). Specially, the light intensity 1000 μmol/m^2^•s was significantly lower (P<0.05) than the rest of moderate light intensity treatments (i.e., 1500, 2000 and 2500 μmol/m^2^•s) (Table 3).

**Table 3:** **Mean values** ± **SD of the nutrient removal efficiency and the economic efficiency under various light intensities by red light wavelength**

**Items**	**Light intensity (μmol/m**^**2**^•**s)**	**Nutrient removal efficiency (%)**			**Economic efficiency (USD)**		
		**COD**	**TN**	**TP**	**COD**	**TN**	**TP**
High C loading	500	7.08^d^±1.34	10.97^d^±1.53	7.48^d^±0.76	12.81^b^±2.54	19.86^b^±3.30	13.54^b^±1.72
	1000	47.58^c^±4.87	45.77^c^±9.69	29.18^c^±6.82	39.52^a^±7.63	38.02^a^±5.38	24.24^a^±4.07
	1500	67.49^b^±8.25	58.64^b^±10.75	41.32^b^±8.80	29.68^a^±4.32	25.79^a^±4.61	18.17^a^±3.81
	2000	78.87^a^±9.49	74.93^a^±8.34	55.83^a^±10.65	27.34^a^±3.75	25.98^a^±3.539	19.35^a^±4.78
	2500	82.12^a^±11.28	76.04^a^±8.39	57.88^a^±7.43	16.16^b^±3.05	14.96^b^±4.19	11.39^b^±2.18
	3000	8.31^d^±0.85	10.84^d^±1.42	8.37^d^±1.13	1.36^c^±0.38	1.78^c^±0.29	1.73^c^±0.43
High N loading	500	8.16^d^±1.78	11.24^d^±1.23	9.26^d^±1.33	14.77^b^±2.31	20.34^b^±1.04	16.76^b^±2.37
	1000	47.45^c^±7.56	44.33^c^±7.77	29.02^c^±6.07	39.41^a^±2.71	36.82^a^±3.10	24.11^a^±2.74
	1500	68.70^b^±8.44	68.50^b^±10.66	39.17^b^±6.54	30.21^a^±2.84	30.12^a^±2.11	17.23^a^±1.92
	2000	80.77^a^±7.49	78.87^a^±7.15	52.93^a^±8.29	28.00^a^±3.09	27.34^a^±3.21	18.35^a^±2.38
	2500	81.90^a^±8.26	81.16^a^±7.04	52.72^a^±7.79	16.11^b^±2.75	15.97^b^±2.59	10.37^b^±2.48
	3000	8.93^d^±1.32	10.25^d^±1.19	8.91^d^±1.39	1.46^c^±0.34	1.68^c^±0.27	1.46^c^±0.15

a, b, c, d: Values with different superscript letters in the same column indicate significant differences at P = 0.05, according to Duncan’s multiple range tests.

### Economic efficiencies under various light intensities

The coefficient of cost per unit, *k*, in Eq. 2 was calculated to be 969.3×10^-4^ USD/kW•h based on the prices in Shanghai City. The energy consumptions of the red light LED for various light intensity treatments were showed in Table 2. The optimal experimental illumination time was determined as 120 h since the maximum microalga DW was achieved during 120 h to 192 h while the highest nutrient removal efficiency was achieved at 120 h. Then, the actual illumination time was 60 h according to the 12-hour light–dark cycle. Therefore, the mean values of economic efficiencies of nutrient removal efficiencies under various light intensities by red light wavelength were demonstrated in Table 3 according to the calculation of Eq. 2.

The economic efficiencies of COD, TN, and TP removal effect demonstrated similar variation trends, and these variations under both high C and N loading treatments were similar. The economic efficiencies of nutrient removal under 500 and 3000 μmol/m^2^•s light intensity was significantly lower (P<0.05) than those of the other treatments since the nutrient removal efficiencies of them were much lower than the others (Table 3). Referring to the treatments at moderate light intensities (1000, 1500, 2000 and 2500 μmol/m^2^•s), there were no significant differences (P>0.05) among the light intensity treatments under 1000, 1500 and 2000 μmol/m^2^•s, while they were significantly higher (P<0.05) than the light intensity treatments under 2500 μmol/m^2^•s (Table 3).

## Discussion

The microalgae reproduction capacity was largely dependent on the characteristic of light wavelength. The microalgal green pigment chlorophyll could absorb the red light wavelength more efficiently than other light wavelength [21]. However, the wavelengths of blue (460 nm to 470 nm) (Table 1), which was characterized by shorter wavelengths, had a much higher probability of striking the light harvesting complex at its peak electrical energy, resulting in too much energy for photosynthesis, which inevitably caused photoinhibition [27]. So the chlorophyll pigment of *C*. *vulgaris* was not good at absorbing blue light wavelength [28]. On the other hand, the red light wavelength was able to avoid photoinhibition since it had relatively longer wavelength [21]. Particularly, the wavelength of white light (380 nm to 670 nm) is a combination of the red wavelength and other growth-inefficient wavelengths (Table 1). Therefore, the DW of white showed a value between red and yellow.

The results about nutrient removal efficiency to various light wavelengths were agreed with the microalgae DW variation mentioned above. The *C*. *vulgaris* reproduction requires abundant nutriment for the synthesis process of nucleic acid, phospholipid and protein [10,31]. Therefore, the phosphorus in the synthetic wastewater was removed by the assimilation effects of *C*. *vulgaris* cells [31]; while the nitrogen was removed in the form of organic nitrogen during the synthesis process of *C*. *vulgaris* cells [10]. The microalgae green pigment chlorophyll in the *C*. *vulgaris* could absorb the red light wavelength high efficiently for the process of photosynthesis, whereas the other light wavelengths could only be partially absorbed. The LED red light wavelength could enhance photosystem II relative to photosystem I [21]. The white light wavelength represented a medium nutrient removal effects, ranking between the red and yellow light. This was because the emission spectrum band of the white light wavelength nearly completely covered that of the red light wavelength (Table 1) and thus, the white wavelength resulted in a combination of the nutrient removal effects of the entire light spectrum.

Under moderate light intensities (1000, 1500, 2000 and 2500 μmol/m^2^•s), in general, the microalgae DW increased quickly from 0 h to 120 h (Figure 1). It was because that the period from 24 h to 124 h served as the logarithmic phase, in which the microalgae cells growth very fast due to the nutrients in the wastewater were sufficient and the metabolic waste of microalgae had not accumulated richly yet [33,34]. Then, the DW nearly remained unchanged from 120 h to 192 h served as the stable phase. In this period, the microalgae reproduction process slowed down compared to the logarithmic phase due to the nutrient depletion and toxic metabolic product accumulation [33]. Finally, the DW decreased from 192 h to 240 h served as the decline phase, in which the available nutrients and resources were soon depleted and the waste products had already accumulated richly [27,33]. Furthermore, the maximum microalga DW was achieved with the light intensity 2500 μmol m^−2^ s^−1^ during 120 h to 192 h experimental illumination time.

The nutrient efficiency increased from 24 h to 120 h and then decreased, under the moderate light intensity (i.e., 1000, 1500, 2000 and 2500 μmol m^−2^ s^−1^). The highest nutrient removal efficiency was achieved at 2500 μmol m^−2^ s^−1^ at 120 h (Figures 2, 3 and 4), which, interestingly, occurred at the start of the logarithmic phase (120 h to 192 h) as illustrated in Figure 1, which is when the maximum DW of *C*. *vulgaris* was obtained and sustained [33,34]. These indicated that the effects of nutrient removal were attributed to the microalgae assimilation process. The microalgae cells assimilated abundant carbon, nitrogen and phosphorus nutrient elements from wastewater for nucleic acid, phospholipid and protein synthesis [31]. Nitrogen was removed mainly in the form of ammonia, as well as organic nitrogen [10]. Furthermore, the highest nutrient removal efficiency was achieved with the light intensity 2500 μmol m^−2^ s^−1^ at 120 h experimental illumination time.

Adequate illumination intensity was essential to microalgae cultures and metabolism [10,31]. Higher light intensities provided sufficient light to promote the reproduction process of microalgae [29], which allowed the microalgae to absorb carbon, nitrogen and phosphorous from the synthetic wastewater as nutrient sources for its own assimilation [25,26]. Therefore, under both high C and N loading, the optimal light intensity in terms of nutrient removal efficiency was 2500 and 2000 μmol/m^2^•s. The lower light intensities treatments consumed less power, although higher light intensity treatment achieved relatively high nutrient removal effect (Table 1). So the optimal light intensity in terms of economic efficiency of nutrient removal was 1000, 1500 and 2000 μmol/m^2^•s, under both high C and N loading. Generally speaking, taking both nutrient removal effects and economic efficiency into account, the optimum light intensity was deemed to be 2000 μmol/m^2^•s.

## Conclusions

The red light was found to be the optimal wavelength for both *C*. *vulgaris* microalgae DW and nutrient removal efficiency. The various nutrient removals and economic efficiencies represented similar variation trends, and these variations under both high C and N loading treatments were similar too. Furthermore, the optimal light intensity in terms of nutrient removal efficiency was 2500 and 2000 μmol/m^2^•s, while in terms of economic efficiency was 1000, 1500 and 2000 μmol/m^2^•s. Therefore, the optimum light intensity was deemed to be 2000 μmol/m^2^•s. In addition, the optimal experimental illumination time was determined as 120 h.

## Competing interests

The authors declare that they have no competing interests.

## Authors’ contributions

Zhigang Ge, Hui Zhang, Yuejin Zhang, Cheng Yan and Yongjun Zhao participated in the design of the study and performed the statistical analysis. Zhigang Ge, Hui Zhang and Yuejin Zhang carried out the experimental studies. Cheng Yan and Yongjun Zhao helped to draft the manuscript. All authors read and approved the final manuscript.
